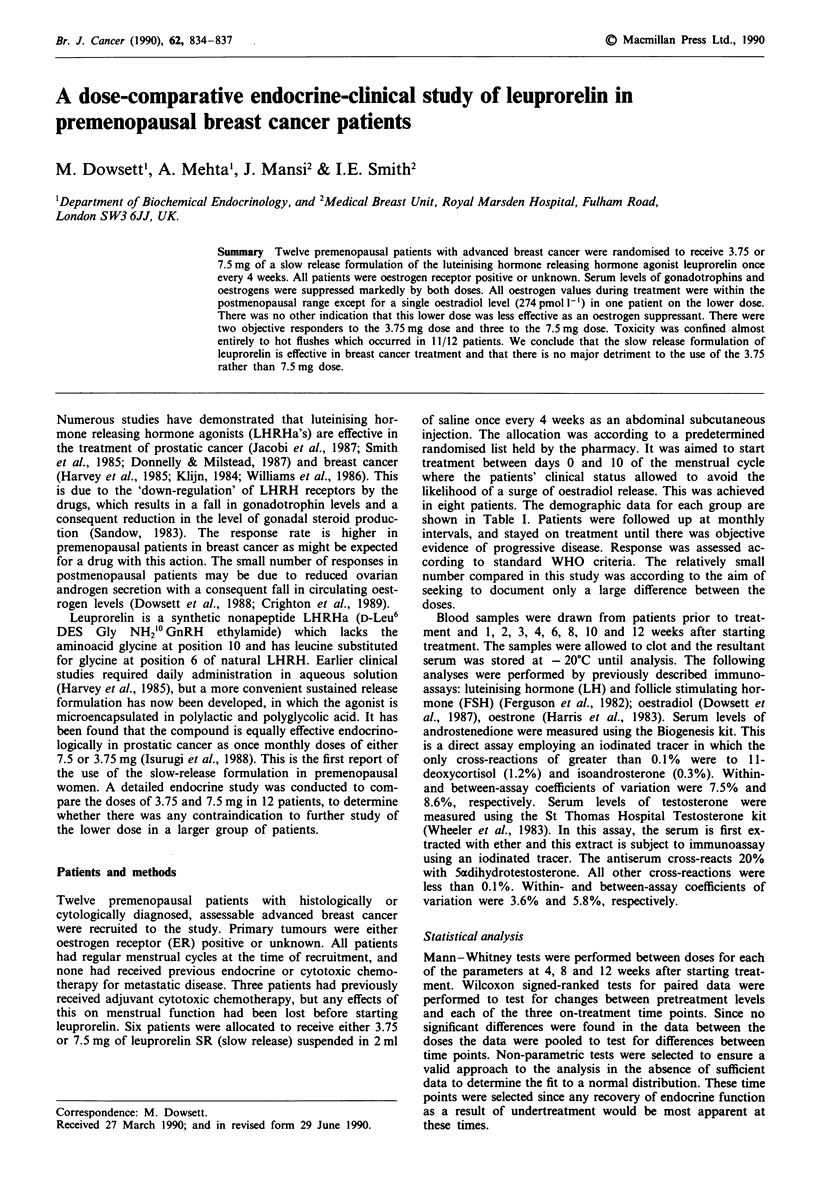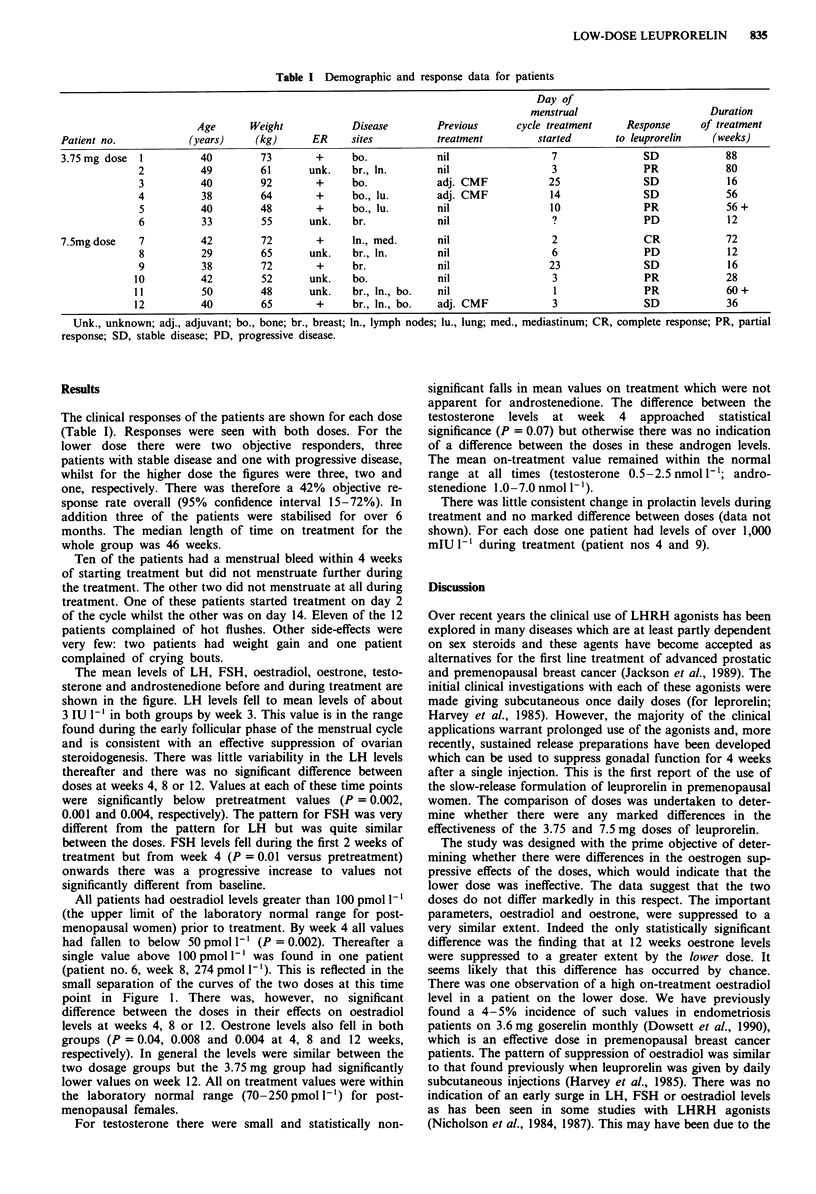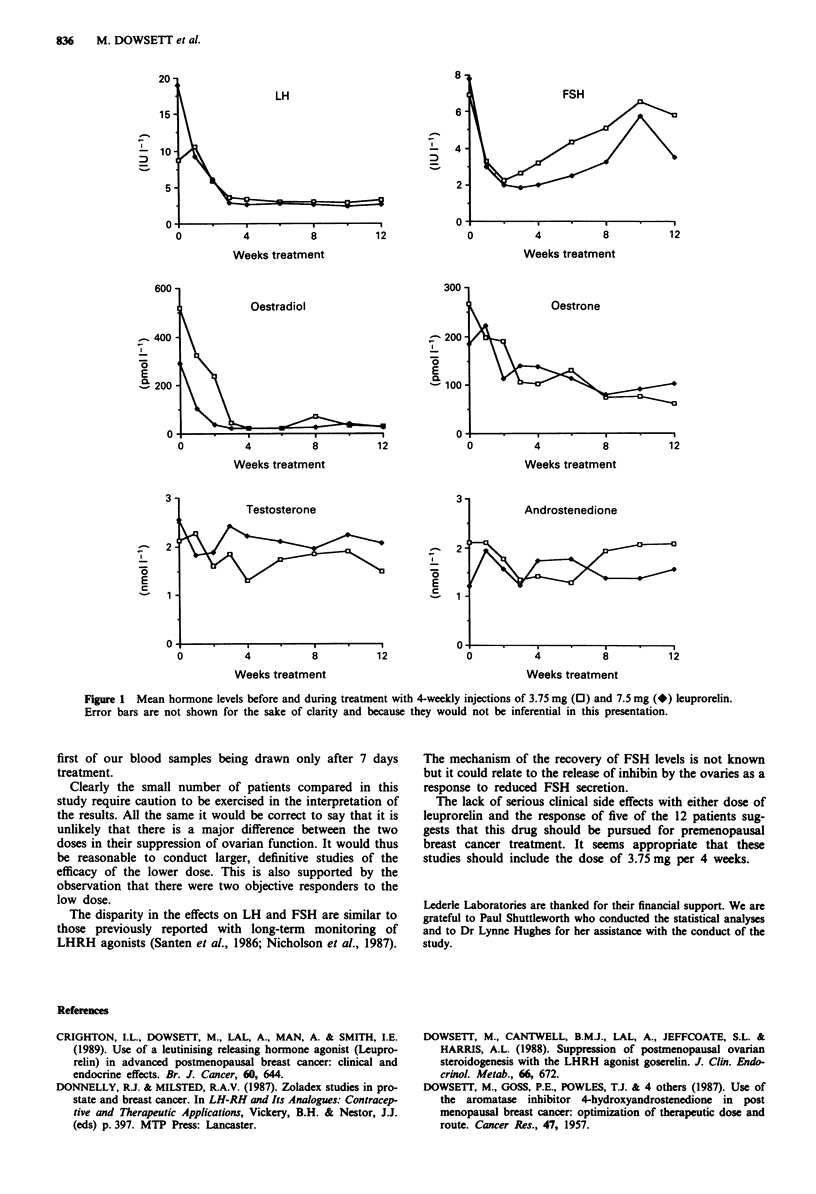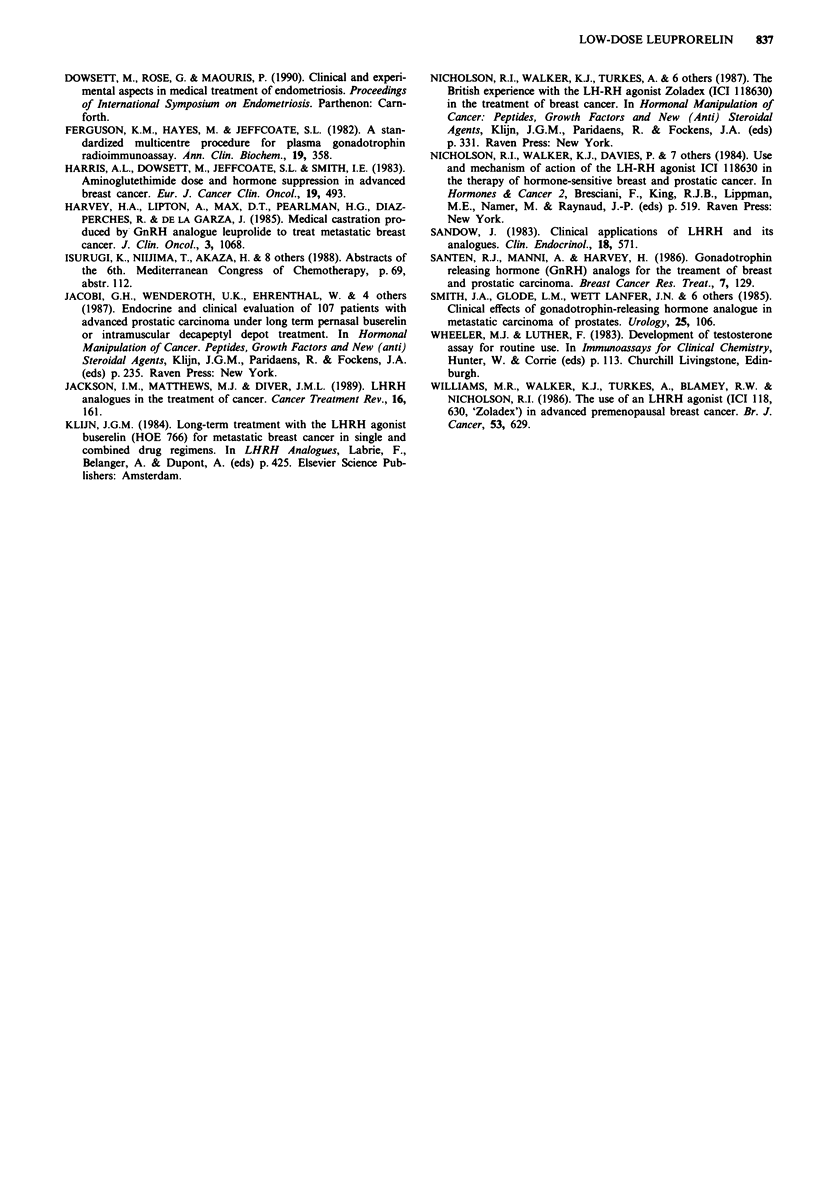# A dose-comparative endocrine-clinical study of leuprorelin in premenopausal breast cancer patients.

**DOI:** 10.1038/bjc.1990.388

**Published:** 1990-11

**Authors:** M. Dowsett, A. Mehta, J. Mansi, I. E. Smith

**Affiliations:** Department of Biochemical Endocrinology, Royal Marsden Hospital, London, UK.

## Abstract

Twelve premenopausal patients with advanced breast cancer were randomised to receive 3.75 or 7.5 mg of a slow release formulation of the luteinising hormone releasing hormone agonist leuprorelin once every 4 weeks. All patients were oestrogen receptor positive or unknown. Serum levels of gonadotrophins and oestrogens were suppressed markedly by both doses. All oestrogen values during treatment were within the postmenopausal range except for a single oestradiol level (274 pmol l-1) in one patient on the lower dose. There was no other indication that this lower dose was less effective as an oestrogen suppressant. There were two objective responders to the 3.75 mg dose and three to the 7.5 mg dose. Toxicity was confined almost entirely to hot flushes which occurred in 11/12 patients. We conclude that the slow release formulation of leuprorelin is effective in breast cancer treatment and that there is no major detriment to the use of the 3.75 rather than 7.5 mg dose.


Br. J. Cancer (1990), 62, 834-837 © Macmillan Press Ltd., 1990

### A dose-comparative endocrine-clinical study of leuprorelin in premenopausal breast cancer patients

M. Dowsett[1], A. Mehta[1], J. Mansi[2] & I.E. Smith[2]

[1]*Department of Biochemical Endocrinology, and* [2]*Medical Breast Unit, Royal Marsden Hospital, Fulham Road, London SW3 6JJ, UK.*

**Summary** Twelve premenopausal patients with advanced breast cancer were randomised to receive 3.75 or 7.5 mg of a slow release formulation of the luteinising hormone releasing hormone agonist leuprorelin once every 4 weeks. All patients were oestrogen receptor positive or unknown. Serum levels of gonadotrophins and oestrogens were suppressed markedly by both doses. All oestrogen values during treatment were within the postmenopausal range except for a single oestradiol level (274 pmol $l^{-1}$) in one patient on the lower dose. There was no other indication that this lower dose was less effective as an oestrogen suppressant. There were two objective responders to the 3.75 mg dose and three to the 7.5 mg dose. Toxicity was confined almost entirely to hot flushes which occurred in 11/12 patients. We conclude that the slow release formulation of leuprorelin is effective in breast cancer treatment and that there is no major detriment to the use of the 3.75 rather than 7.5 mg dose.

Numerous studies have demonstrated that luteinising hormone releasing hormone agonists (LHRHa's) are effective in the treatment of prostatic cancer (Jacobi *et al.*, 1987; Smith *et al.*, 1985; Donnelly & Milstead, 1987) and breast cancer (Harvey *et al.*, 1985; Klijn, 1984; Williams *et al.*, 1986). This is due to the 'down-regulation' of LHRH receptors by the drugs, which results in a fall in gonadotrophin levels and a consequent reduction in the level of gonadal steroid production (Sandow, 1983). The response rate is higher in premenopausal patients in breast cancer as might be expected for a drug with this action. The small number of responses in postmenopausal patients may be due to reduced ovarian androgen secretion with a consequent fall in circulating oestrogen levels (Dowsett *et al.*, 1988; Crighton *et al.*, 1989).

Leuprorelin is a synthetic nonapeptide LHRHa (D-Leu$^6$ DES Gly $NH_2$$^{10}$ GnRH ethylamide) which lacks the aminoacid glycine at position 10 and has leucine substituted for glycine at position 6 of natural LHRH. Earlier clinical studies required daily administration in aqueous solution (Harvey *et al.*, 1985), but a more convenient sustained release formulation has now been developed, in which the agonist is microencapsulated in polylactic and polyglycolic acid. It has been found that the compound is equally effective endocrinologically in prostatic cancer as once monthly doses of either 7.5 or 3.75 mg (Isurugi *et al.*, 1988). This is the first report of the use of the slow-release formulation in premenopausal women. A detailed endocrine study was conducted to compare the doses of 3.75 and 7.5 mg in 12 patients, to determine whether there was any contraindication to further study of the lower dose in a larger group of patients.

### Patients and methods

Twelve premenopausal patients with histologically or cytologically diagnosed, assessable advanced breast cancer were recruited to the study. Primary tumours were either oestrogen receptor (ER) positive or unknown. All patients had regular menstrual cycles at the time of recruitment, and none had received previous endocrine or cytotoxic chemotherapy for metastatic disease. Three patients had previously received adjuvant cytotoxic chemotherapy, but any effects of this on menstrual function had been lost before starting leuprorelin. Six patients were allocated to receive either 3.75 or 7.5 mg of leuprorelin SR (slow release) suspended in 2 ml of saline once every 4 weeks as an abdominal subcutaneous injection. The allocation was according to a predetermined randomised list held by the pharmacy. It was aimed to start treatment between days 0 and 10 of the menstrual cycle where the patients' clinical status allowed to avoid the likelihood of a surge of oestradiol release. This was achieved in eight patients. The demographic data for each group are shown in Table I. Patients were followed up at monthly intervals, and stayed on treatment until there was objective evidence of progressive disease. Response was assessed according to standard WHO criteria. The relatively small number compared in this study was according to the aim of seeking to document only a large difference between the doses.

Blood samples were drawn from patients prior to treatment and 1, 2, 3, 4, 6, 8, 10 and 12 weeks after starting treatment. The samples were allowed to clot and the resultant serum was stored at $-20$°C until analysis. The following analyses were performed by previously described immunoassays: luteinising hormone (LH) and follicle stimulating hormone (FSH) (Ferguson *et al.*, 1982); oestradiol (Dowsett *et al.*, 1987), oestrone (Harris *et al.*, 1983). Serum levels of androstenedione were measured using the Biogenesis kit. This is a direct assay employing an iodinated tracer in which the only cross-reactions of greater than 0.1% were to 11-deoxycortisol (1.2%) and isoandrosterone (0.3%). Within- and between-assay coefficients of variation were 7.5% and 8.6%, respectively. Serum levels of testosterone were measured using the St Thomas Hospital Testosterone kit (Wheeler *et al.*, 1983). In this assay, the serum is first extracted with ether and this extract is subject to immunoassay using an iodinated tracer. The antiserum cross-reacts 20% with 5αdihydrotestosterone. All other cross-reactions were less than 0.1%. Within- and between-assay coefficients of variation were 3.6% and 5.8%, respectively.

### *Statistical analysis*

Mann-Whitney tests were performed between doses for each of the parameters at 4, 8 and 12 weeks after starting treatment. Wilcoxon signed-ranked tests for paired data were performed to test for changes between pretreatment levels and each of the three on-treatment time points. Since no significant differences were found in the data between the doses the data were pooled to test for differences between time points. Non-parametric tests were selected to ensure a valid approach to the analysis in the absence of sufficient data to determine the fit to a normal distribution. These time points were selected since any recovery of endocrine function as a result of undertreatment would be most apparent at these times.

Correspondence: M. Dowsett.
Received 27 March 1990; and in revised form 29 June 1990.

**Table I** Demographic and response data for patients

| Patient no. | | Age (years) | Weight (kg) | ER | Disease sites | Previous treatment | Day of menstrual cycle treatment started | Response to leuprorelin | Duration of treatment (weeks) |
|---|---|---|---|---|---|---|---|---|---|
| 3.75 mg dose | 1 | 40 | 73 | + | bo. | nil | 7 | SD | 88 |
| | 2 | 49 | 61 | unk. | br., ln. | nil | 3 | PR | 80 |
| | 3 | 40 | 92 | + | bo. | adj. CMF | 25 | SD | 16 |
| | 4 | 38 | 64 | + | bo., lu. | adj. CMF | 14 | SD | 56 |
| | 5 | 40 | 48 | + | bo., lu. | nil | 10 | PR | 56 + |
| | 6 | 33 | 55 | unk. | br. | nil | ? | PD | 12 |
| 7.5mg dose | 7 | 42 | 72 | + | ln., med. | nil | 2 | CR | 72 |
| | 8 | 29 | 65 | unk. | br., ln. | nil | 6 | PD | 12 |
| | 9 | 38 | 72 | + | br. | nil | 23 | SD | 16 |
| | 10 | 42 | 52 | unk. | bo. | nil | 3 | PR | 28 |
| | 11 | 50 | 48 | unk. | br., ln., bo. | nil | 1 | PR | 60 + |
| | 12 | 40 | 65 | + | br., ln., bo. | adj. CMF | 3 | SD | 36 |

Unk., unknown; adj., adjuvant; bo., bone; br., breast; ln., lymph nodes; lu., lung; med., mediastinum; CR, complete response; PR, partial response; SD, stable disease; PD, progressive disease.

### Results

The clinical responses of the patients are shown for each dose (Table I). Responses were seen with both doses. For the lower dose there were two objective responders, three patients with stable disease and one with progressive disease, whilst for the higher dose the figures were three, two and one, respectively. There was therefore a 42% objective response rate overall (95% confidence interval 15–72%). In addition three of the patients were stabilised for over 6 months. The median length of time on treatment for the whole group was 46 weeks.

Ten of the patients had a menstrual bleed within 4 weeks of starting treatment but did not menstruate further during the treatment. The other two did not menstruate at all during treatment. One of these patients started treatment on day 2 of the cycle whilst the other was on day 14. Eleven of the 12 patients complained of hot flushes. Other side-effects were very few: two patients had weight gain and one patient complained of crying bouts.

The mean levels of LH, FSH, oestradiol, oestrone, testosterone and androstenedione before and during treatment are shown in the figure. LH levels fell to mean levels of about 3 IU $l^{-1}$ in both groups by week 3. This value is in the range found during the early follicular phase of the menstrual cycle and is consistent with an effective suppression of ovarian steroidogenesis. There was little variability in the LH levels thereafter and there was no significant difference between doses at weeks 4, 8 or 12. Values at each of these time points were significantly below pretreatment values ($P = 0.002$, 0.001 and 0.004, respectively). The pattern for FSH was very different from the pattern for LH but was quite similar between the doses. FSH levels fell during the first 2 weeks of treatment but from week 4 ($P = 0.01$ versus pretreatment) onwards there was a progressive increase to values not significantly different from baseline.

All patients had oestradiol levels greater than 100 pmol $l^{-1}$ (the upper limit of the laboratory normal range for postmenopausal women) prior to treatment. By week 4 all values had fallen to below 50 pmol $l^{-1}$ ($P = 0.002$). Thereafter a single value above 100 pmol $l^{-1}$ was found in one patient (patient no. 6, week 8, 274 pmol $l^{-1}$). This is reflected in the small separation of the curves of the two doses at this time point in Figure 1. There was, however, no significant difference between the doses in their effects on oestradiol levels at weeks 4, 8 or 12. Oestrone levels also fell in both groups ($P = 0.04$, 0.008 and 0.004 at 4, 8 and 12 weeks, respectively). In general the levels were similar between the two dosage groups but the 3.75 mg group had significantly lower values on week 12. All on treatment values were within the laboratory normal range (70–250 pmol $l^{-1}$) for postmenopausal females.

For testosterone there were small and statistically non-significant falls in mean values on treatment which were not apparent for androstenedione. The difference between the testosterone levels at week 4 approached statistical significance ($P = 0.07$) but otherwise there was no indication of a difference between the doses in these androgen levels. The mean on-treatment value remained within the normal range at all times (testosterone 0.5–2.5 nmol $l^{-1}$; androstenedione 1.0–7.0 nmol $l^{-1}$).

There was little consistent change in prolactin levels during treatment and no marked difference between doses (data not shown). For each dose one patient had levels of over 1,000 mIU $l^{-1}$ during treatment (patient nos 4 and 9).

### Discussion

Over recent years the clinical use of LHRH agonists has been explored in many diseases which are at least partly dependent on sex steroids and these agents have become accepted as alternatives for the first line treatment of advanced prostatic and premenopausal breast cancer (Jackson *et al.*, 1989). The initial clinical investigations with each of these agonists were made giving subcutaneous once daily doses (for leprorelin; Harvey *et al.*, 1985). However, the majority of the clinical applications warrant prolonged use of the agonists and, more recently, sustained release preparations have been developed which can be used to suppress gonadal function for 4 weeks after a single injection. This is the first report of the use of the slow-release formulation of leuprorelin in premenopausal women. The comparison of doses was undertaken to determine whether there were any marked differences in the effectiveness of the 3.75 and 7.5 mg doses of leuprorelin.

The study was designed with the prime objective of determining whether there were differences in the oestrogen suppressive effects of the doses, which would indicate that the lower dose was ineffective. The data suggest that the two doses do not differ markedly in this respect. The important parameters, oestradiol and oestrone, were suppressed to a very similar extent. Indeed the only statistically significant difference was the finding that at 12 weeks oestrone levels were suppressed to a greater extent by the *lower* dose. It seems likely that this difference has occurred by chance. There was one observation of a high on-treatment oestradiol level in a patient on the lower dose. We have previously found a 4–5% incidence of such values in endometriosis patients on 3.6 mg goserelin monthly (Dowsett *et al.*, 1990), which is an effective dose in premenopausal breast cancer patients. The pattern of suppression of oestradiol was similar to that found previously when leuprorelin was given by daily subcutaneous injections (Harvey *et al.*, 1985). There was no indication of an early surge in LH, FSH or oestradiol levels as has been seen in some studies with LHRH agonists (Nicholson *et al.*, 1984, 1987). This may have been due to the

**Figure 1** Mean hormone levels before and during treatment with 4-weekly injections of 3.75 mg (□) and 7.5 mg (◆) leuprorelin. Error bars are not shown for the sake of clarity and because they would not be inferential in this presentation.

first of our blood samples being drawn only after 7 days treatment.

Clearly the small number of patients compared in this study require caution to be exercised in the interpretation of the results. All the same it would be correct to say that it is unlikely that there is a major difference between the two doses in their suppression of ovarian function. It would thus be reasonable to conduct larger, definitive studies of the efficacy of the lower dose. This is also supported by the observation that there were two objective responders to the low dose.

The disparity in the effects on LH and FSH are similar to those previously reported with long-term monitoring of LHRH agonists (Santen *et al.*, 1986; Nicholson *et al.*, 1987). The mechanism of the recovery of FSH levels is not known but it could relate to the release of inhibin by the ovaries as a response to reduced FSH secretion.

The lack of serious clinical side effects with either dose of leuprorelin and the response of five of the 12 patients suggests that this drug should be pursued for premenopausal breast cancer treatment. It seems appropriate that these studies should include the dose of 3.75 mg per 4 weeks.

Lederle Laboratories are thanked for their financial support. We are grateful to Paul Shuttleworth who conducted the statistical analyses and to Dr Lynne Hughes for her assistance with the conduct of the study.

### References

CRIGHTON, I.L., DOWSETT, M., LAL, A., MAN, A. & SMITH, I.E. (1989). Use of a leutinising releasing hormone agonist (Leuprorelin) in advanced postmenopausal breast cancer: clinical and endocrine effects. *Br. J. Cancer*, **60**, 644.

DONNELLY, R.J. & MILSTED, R.A.V. (1987). Zoladex studies in prostate and breast cancer. In *LH-RH and Its Analogues: Contraceptive and Therapeutic Applications*, Vickery, B.H. & Nestor, J.J. (eds) p. 397. MTP Press: Lancaster.

DOWSETT, M., CANTWELL, B.M.J., LAL, A., JEFFCOATE, S.L. & HARRIS, A.L. (1988). Suppression of postmenopausal ovarian steroidogenesis with the LHRH agonist goserelin. *J. Clin. Endocrinol. Metab.*, **66**, 672.

DOWSETT, M., GOSS, P.E., POWLES, T.J. & 4 others (1987). Use of the aromatase inhibitor 4-hydroxyandrostenedione in post menopausal breast cancer: optimization of therapeutic dose and route. *Cancer Res.*, **47**, 1957.

DOWSETT, M., ROSE, G. & MAOURIS, P. (1990). Clinical and experimental aspects in medical treatment of endometriosis. *Proceedings of International Symposium on Endometriosis*. Parthenon: Carnforth.

FERGUSON, K.M., HAYES, M. & JEFFCOATE, S.L. (1982). A standardized multicentre procedure for plasma gonadotrophin radioimmunoassay. *Ann. Clin. Biochem.*, **19**, 358.

HARRIS, A.L., DOWSETT, M., JEFFCOATE, S.L. & SMITH, I.E. (1983). Aminoglutethimide dose and hormone suppression in advanced breast cancer. *Eur. J. Cancer Clin. Oncol.*, **19**, 493.

HARVEY, H.A., LIPTON, A., MAX, D.T., PEARLMAN, H.G., DIAZ-PERCHES, R. & DE LA GARZA, J. (1985). Medical castration produced by GnRH analogue leuprolide to treat metastatic breast cancer. *J. Clin. Oncol.*, **3**, 1068.

ISURUGI, K., NIIJIMA, T., AKAZA, H. & 8 others (1988). Abstracts of the 6th. Mediterranean Congress of Chemotherapy, p. 69, abstr. 112.

JACOBI, G.H., WENDEROTH, U.K., EHRENTHAL, W. & 4 others (1987). Endocrine and clinical evaluation of 107 patients with advanced prostatic carcinoma under long term pernasal buserelin or intramuscular decapeptyl depot treatment. In *Hormonal Manipulation of Cancer. Peptides, Growth Factors and New (anti) Steroidal Agents*, Klijn, J.G.M., Paridaens, R. & Fockens, J.A. (eds) p. 235. Raven Press: New York.

JACKSON, I.M., MATTHEWS, M.J. & DIVER, J.M.L. (1989). LHRH analogues in the treatment of cancer. *Cancer Treatment Rev.*, **16**, 161.

KLIJN, J.G.M. (1984). Long-term treatment with the LHRH agonist buserelin (HOE 766) for metastatic breast cancer in single and combined drug regimens. In *LHRH Analogues*, Labrie, F., Belanger, A. & Dupont, A. (eds) p. 425. Elsevier Science Publishers: Amsterdam.

NICHOLSON, R.I., WALKER, K.J., TURKES, A. & 6 others (1987). The British experience with the LH-RH agonist Zoladex (ICI 118630) in the treatment of breast cancer. In *Hormonal Manipulation of Cancer: Peptides, Growth Factors and New (Anti) Steroidal Agents*, Klijn, J.G.M., Paridaens, R. & Fockens, J.A. (eds) p. 331. Raven Press: New York.

NICHOLSON, R.I., WALKER, K.J., DAVIES, P. & 7 others (1984). Use and mechanism of action of the LH-RH agonist ICI 118630 in the therapy of hormone-sensitive breast and prostatic cancer. In *Hormones & Cancer 2*, Bresciani, F., King, R.J.B., Lippman, M.E., Namer, M. & Raynaud, J.-P. (eds) p. 519. Raven Press: New York.

SANDOW, J. (1983). Clinical applications of LHRH and its analogues. *Clin. Endocrinol.*, **18**, 571.

SANTEN, R.J., MANNI, A. & HARVEY, H. (1986). Gonadotrophin releasing hormone (GnRH) analogs for the treament of breast and prostatic carcinoma. *Breast Cancer Res. Treat.*, **7**, 129.

SMITH, J.A., GLODE, L.M., WETT LANFER, J.N. & 6 others (1985). Clinical effects of gonadotrophin-releasing hormone analogue in metastatic carcinoma of prostates. *Urology*, **25**, 106.

WHEELER, M.J. & LUTHER, F. (1983). Development of testosterone assay for routine use. In *Immunoassays for Clinical Chemistry*, Hunter, W. & Corrie (eds) p. 113. Churchill Livingstone, Edinburgh.

WILLIAMS, M.R., WALKER, K.J., TURKES, A., BLAMEY, R.W. & NICHOLSON, R.I. (1986). The use of an LHRH agonist (ICI 118, 630, 'Zoladex') in advanced premenopausal breast cancer. *Br. J. Cancer*, **53**, 629.